# Sustainable solid waste management Measures in Tanzania: an exploratory descriptive case study among vendors at Majengo market in Dodoma City

**DOI:** 10.1186/s12889-020-08670-0

**Published:** 2020-07-08

**Authors:** Kepha Nyampundu, William J. S. Mwegoha, Walter C. Millanzi

**Affiliations:** 1 Department of Environmental and Engineering Management, College of Earth Sciencess, The University of Dodoma, Dodoma, Tanzania; 2 College of Health Sciences, The University of Dodoma, Dodoma, Tanzania

**Keywords:** Solid waste, Generation, Rate, Strategies, Market, Management, Vendor, Sustainable

## Abstract

**Background:**

Solid waste management is both an urban and rural problem because every person is considered a producer of wastes. It has been noted to be a global universal issue, which affects every individual, families, communities and governments and thus, needs to be addressed through sustainable strategies. This study aimed at characterizing solid wastes; assessing the levels of awareness of vendors on the sustainable solid waste management measures; and identifying techniques used to handle solid waste generated at the Majengo market in Dodoma City, Tanzania.

**Methods:**

The study adopted an exploratory descriptive case study, with a mixed research approaches with a minimum sample of 196 conviniently selected respondents. Semi-structured questionnaires developed by the researcher were the main data collection tools to characterize solid wastes, measure levels of awareness about sustainable solid waste management approaches (SWM) and identify solid waste handling techniques among vendors at the market. Quantitative and Qualitative data were analyzed by using the Statistical Product for Social Sciences version 23 and thematic analysis respectively.

**Results:**

Findings showed that 55% of vendors were males. Majority of the sampled vendors (56%) were not aware of SWM. On the other hand, crops/food and animal product remains were reported to be the most generated solid wastes (94.4%) with the rate of > 2 tons/day equivalent to 72.4% of the overall solid waste generation at the market. SWM services were reported to be provided by City council (85.7%) with the main equipment/tool used to store solid wastes (SW) being containers without lids (88.3%). The dumpsite was the main site for SW disposal (80.1%). Nevertheless, 92.9% of the sampled vendors reported that SWM strategies were there at the market though not adhered to accordingly.

**Conclusion:**

Vendors were not aware of sustainable solid waste management measures existing at the market. However, vendors’ education levels and the duration of doing business at the market were related to their levels of awareness on SWM (*p* < 0.05). The SWM measures were found to exist at the market, however, they were not sustainable because it was reported that they were ineffectively and inefficiently used to control SW generation, collection, storage, and disposal. There is a need of regular awareness-raising activities about sustainable SWM measures among vendors. Moreover, city council and market authorities need to have sustainable and scheduled implementation, supervision, monitoring and evaluation of SWM measures to maintain the management of solid wastes at Majengo market premises.

## Background

The fundamental global environmental issue in both industrial and developing countries is how to best identify and manage waste [1]. Solid waste management has been noted to be a global universal issue, which affects every individual and government [2]. As urbanization continues to take place, the management of solid wastes (MSW) poses major public health and environmental problem particularly in urban areas all over the world [3]. The UN [4] edition of world urbanization prospects noted that about 90% of urban growth will take place in Africa and Asian countries due to the migration of people from rural to urban areas.

Currently, the remarkably progressive and rapid urbanization has overwhelmed the global, regional, national, and local authorities, particularly of the developing countries to plan for it [1]. This has led to the constrain of quality and efficient institutional, and organizational structures that in turn affects the provision of various public services, and to plan for sustainable infrastructures in the favor of solid waste management and characterization [2].

Solid waste is described as any discarded materials that arise from human activities and are not free-flowing including bottles, metals and plastic scrapers, garbage, papers, glasses, and food/animal product, disposable carrying bags, woods, and or malfunctioned electronic devices [4–6]. They can be classified as domestic, commercial/market, industrial, hospital, or street sweepings. Furthermore, municipal solid waste management is a multi-disciplinary natural and social sciences approach, which describes the process of controlling the production, storage, collection, transportation, processing, and disposal of municipal solid wastes including those, which are produced in the market places [7].

Owing to the growing population, the quantity and variety of solid wastes from domestic, social, industrial activities, the development of technologies, agricultural activities, livestock keeping, and commercials, continue to increase in most African countries [5]. Individuals, families, communities, municipalities, cities, markets, private and government offices, and conference rooms are responsible for public health and safety [6]. Careful sustainable management of solid wastes can prevent damage done to the environment and conserve scarce resources. However; solid waste disposal and management are given low priority, particularly in most developing countries.

Solid waste disposal and management is both an urban and rural problem as every person is considered as a producer of wastes and thus a contributor to the problem [8]. In the current study, solid waste management was defined as any sustainable and integrated environmentally cleanliness approach, which aimed at making the environment specifically at market premises, clean and neat. One of the global agendas is to increase awareness of the environmental issues among people to address the problem of solid wastes. Municipal solid waste management stakeholders are mandated to take collective action under the decentralized systems to achieve municipal sustainable solid waste service provision especially in market places [8, 9].

The decentralized systems of solid waste management include local authorities and the abilities of municipalities to develop, execute, and evaluate market-based policies, standards, and rules, which aim at managing wastes. Moreover, local authorities have the roles of the presence of effective and efficient solid waste collection, storage, and transportation and disposal centers including solid waste collection and transportation utensils or equipment such as bins and trucks. They also play a key role in establishing solid waste collection, transportation, and disposal schedule from their respective areas to the treatment and dumping sites.

However, the presence of bottles, metals and plastic scrapers, garbage, papers, glasses, and food/animal product, disposable carrying bags, woods, and or malfunctioned electronic devices in the environment, particularly in the market places, would indicate the inadequacy of SWM strategies implementation. Solid waste management strategies as defined in the current study are approaches used to manage wastes generation, storage, collection, transportation, treatment, and disposal at the final dumping place. They include the presence of market-based SWM leadership, guidelines, rules, and standards, equipment such as bins and trucks, storage and collection center and a schedule for SW disposal. The systemic failure of policymakers and local authorities to identify the most sustainable SWM approach to meet the environmental and socio-economic aspirations, complex waste operations and also expensive running costs of urban solid waste management, has led to the improper SWM [2, 7, 8].

Consequently, a huge uncollected solid waste, especially in market places, cause clogging of drains, flooding, health problems caused by vectors that transmit diseases, harming animals and people, who consume hazardous waste unknowingly, environmental pollution, air pollution from airborne particles, and affecting social-economic development such as through diminished tourism, particularly in developing countries.

In many developing countries, the lack of viable alternatives to handle market waste management efficiently and effectively to prevent environmental pollutions is still a challenge [2]. The main sources of pollution include municipal wastewater from leaking sewage systems (the most serious), heavy smoke and chemical emissions from agricultural activities, and air pollution and vehicle scraps from transportation activities, electronic and non-electronic scraps from commercial/market activities, and noises, gaseous emissions from industrial activities and leachates from dumpsites [4].

Tanzania like other developing countries faces a serious concern and challenges on solid waste management, which is more pronounced in the commercial and market places where most people visit to sell or buy goods without necessary infrastructures and quality social services [10, 11]. The most common market solid wastes in the country include among others; bottles, metals, garbage, papers, plastics, glasses, and food/animal product remains [2].

Rapid urban development in Tanzania and Dodoma city, in particular, has brought significant benefits to people such as employment and socio-economic development. However, the status has caused environmental problems especially in market places that endanger public health. Moreover, the limited sorting process of solid wastes at the source that leads to improper collection, storage, transportation, treatment, and final disposal at the dumping areas is experienced in the country [9].

This an indicator of unsatisfactory SWM in the country that might be attributed by either lack of enforcement of environmental laws, regulations, or the awareness levels, unwillingness of the urban dwellers and vendors to participate in SWM and or pay for the waste management services [9, 10]. Based on that solid waste management in cities and towns might not be as effective to manage ever-increasing volume and variety of waste, solid waste collection, storage, and transportation and disposal system as it is required.

The Environment Management Act (EMA) No. 20 of 2004, and the National Environment Management Council (NEMC) Act No. 19 of 1983, is in practice in Tanzania to make sure that environmental conservation and sanitation are maintained. However, municipal solid waste management has been commonly the largest single budget item for communities, industries, institutions, and business areas such as in markets [9]. In the face of that, approximately more than 10,000 tons of municipal solid wastes are generated per day with a rate ranging from 0.1 to 1.0 kg/cap/day Countrywide, whereby [9].

Nevertheless, the National Environment Statistics reported that 2,101,500 tones of wastes, for example, are generated in Tanzania mainland regions including Arusha, Songwe, Dodoma, Mwanza, Songea, and Rukwa. It has been reported that out of which, 1,196,900 tones (57%) of the total generated waste in those regions came from the households, 279,400 tones (13.3%) construction wastes and the remained 625,200 tones (29.7%) wastes from other sources including market places.

Yet, despite the decentralization of authorities to municipal councils, city councils and market leadership by the government of Tanzania and involvement of community-based solid waste collection teams to assure solid waste management, data show that 80 to 90% of the total solid waste generated in urban areas daily is not collected [9]. On the other hand, about 60% of the daily domestic generated solid waste is disposed of by burning and composited to be used as manure in farms or burying [9].

The uncollected solid waste stays longer in one area and spread all over the environment when the wind comes or birds and animal feed them as food leading to environmental, air and water pollution. Additionally, the country’s trend of solid wastes generation increased from 251 tons in 2010 to 278 tons per day in 2011. It was estimated that, of the total population, each person was producing an average solid waste of about 0.5 kg to 0.8 kg per day in 2011 including those produced at the market places [12].

Various solid waste management policies and guidelines on solid waste management including the National Environmental Policy (NEP) of 1997, Environment Management Act (EMA) No. 20 of 2004 and National Environment Management Council (NEMC) Act No. 19 of 1983 have made to control environmental sanitation in Tanzania. The NEP emphasizes sustainable environment management, security, and equitable use of resources, raising public awareness, and promoting individual and community participation in environmental management. The policy is still implemented though there is a scarcity of information on its effectiveness about waste management on the market places. On this basis, continued research is necessary.

The National Environment Management Council (NEMC) Act mandates the environmental management Authorities to ensure a clean, safe, and healthy environment for people in Tanzania through the coordination of environmental management activities. Moreover, it emphasizes awareness rising on waste management among people in all geographical locations, enforcement of the environment management policy, its assessment, monitoring, and guide for planning and undertaking environmental research programs, projects, and activities in the country.

Issues on enforcement of the waste management policy, its assessment, and monitoring are being implemented. However, many urban authorities are facing challenges on how to manage solid wastes at the market places due to the rapid urban growth coupled with the scarcity of funds [11]. In this regard, little is still known about the level and role of awareness rising programs stipulated in the EMA on waste management among vendors and other people in the market and researches are being implemented and yield positive results in managing SW.

On the other hand, The Environment Management Act (EMA) requires authorities to put more efforts to promote the state of the actual environment and its associated future threats such as any emission to air, land or water and the storage and disposal of non-hazardous and hazardous wastes (waste segregation). The Act requires the appointed sectoral, regional and the District Environmental management coordinators to enforce environmental management policy to their geographical locations, implement, monitor, and evaluate it for its effectiveness. However, there has been a lack of adequate and sound reports as well as research data on the effectiveness of the Act implementation on environment management (waste management), particularly in market places.

Literature and reports [1, 2, 8, 11, 13–15] has found that there is low market solid wastes management due to amongst others such as financial resources and infrastructures, sensitization and awareness-raising about solid waste management among people. Levels of awareness on SWM among people including vendors have been observed to influence their practices on proper waste segregation, reduction, and recycling. However, available information from the pieces of the literature revealed little evidence on the influence of awareness on sustainable solid waste management in terms of reuse and disposal.

This probably might lead to the failure of the council and market authorities to make the best timely mechanisms to control and manage SW generation, storage, collection, transportation, treatment, and disposal. Based on that, SW such as papers, food, and animal remains, disposable carrying bags and other related materials are sometimes found scattered in the market premises and nearby households by either winds or water flow especially during rainy seasons. This is an indicator that there might be an inadequate implementation of sustainable policy guidelines and approaches in handling solid waste in the market. The government of Tanzania through local government authorities is looking into different approaches and other sustainable solutions to manage solid wastes [14].

Dodoma Region is one of the newly announced and growing city in Tanzania. Dodoma City had a total population of 410,956 people in the 2012 census [10]. The region development vision is ‘promoting Dodoma region such that it becomes a new business hub and growth pole for the East and Central African Economic Region’. This is an added indicator to the urbanization, which continues to take place in the region that, the generation of solid wastes would continue to be high both in volume and variety in its environment including market places.

The councils through Environment management departments use various approaches to manage solid wastes including the decentralization of SWM authorities to the market centers, employment of community-based SWM teams, and information dissemination about SWM among people who come to purchase goods at the market, development of market-based SWM schedules, standards, and guidelines. However, Dodoma City experienced a serious municipal solid wastes management problem. Solid wastes generated in Dodoma City were estimated to be 305 tons per day. The wastes were mainly from different sources including institutions such as schools/universities (20 tons); shops, hostels, (22 tons); street sweepings, industries, (15 tons); markets (30 tons) and domestic houses waste (218 tons) per day [12].

The capacity of the city to manage solid waste in terms of supplying human resources and non-human resources such as infrastructures (trucks, bins, insecticides, reserve the protected waste storage centers, fumigations) and financial resources was estimated to be 33% (100 tons) while the remaining 67% of the daily-generated solid waste was improperly managed or disposed of [12]. This trend of high volume solid waste generation and improper SWM would, in turn, lead to the cost-fully management of environmental hygiene throughout the city and difficulties in delivering public health services among people particularly in market places to protect them from communicable diseases like cholera, dysentery, malaria, pneumonia, flue, and typhoid.

On the other hand, Dodoma city has three large markets including Saba Saba, Majengo central, and Bonanza markets [16]. Majengo market is located more at the center of the city council and it serves a larger number of people from different places within and outside the city. Moreover, apart from being a place where people visit every day to sell and buy goods, the market serves as a place where people can gather and share the news. The most sold products at the Majengo market include consumables such as foods (packed and unpacked), drinks, and animal products and non-consumables such as clothing, paper bags, customers’ carrying bags of their purchase, technological and electronic equipment, and industrial services. The generated solid wastes came from these sold products, which when the waste collection containers were full, a new visitor would find them scattered all over the market premises such as food shells, husks, food remains, and casings.

The market has its internal leadership, which its leaders are selected amongst vendors themselves whom their role was to lead and manage the market in collaboration with the city council authority. The market leaders have the roles of making sure that waste collection and disposal equipment were enough and available all the time and thus, generated solid wastes by customers, vendors and market visitors were appropriately disposed into them. Moreover, they have the role to enforce environmental sanitation policy set by the council, which required them to catch responsible anyone who was caught violating the market solid waste management rules by having them either pay Tanzanian shillings not less than fifty thousand cash or clean the environment or both of them.

However, the market lack some activities, which in one way or the other would facilitate a sustainable SWM in the market such as scavenging activities whereby people pick through the waste to find potential consumable and non-consumable scraps that could be recycled. High volume and variety of solid wastes generated in the market places is an indicator that there was something wrong either on the level of awareness of vendors on SWM or the compliance of city council and market leadership to implementing sustainable SWM strategies in an attempt to handle and manage solid wastes.

Literatures [3, 12, 14, 16], have been conducted to add new knowledge on the best ways to be employed on SWM across countries and councils as well. Studies on the aspect of assessing how solid waste management authorities and solid waste service providers contributed to the implementation of sustainable solid waste management have been extensively conducted. However, little has been exposed and published on the degree to which the level of awareness vendors has on SWM in market places. Moreover, little is still known on the effectiveness of environmentally Solid Waste Management measures used at Majengo Market in Dodoma City particularly in solid wastes collection, storage, transportation, treatment, and disposal. There is a scarcity of the locally available scholarly works on this topic under study about Dodoma city.

If the status of solid waste generation continues to increase without being sustainably addressed through consistent and effective implementation of the sustainable SWM measures, potential contamination of groundwater sources, organic and inorganic pollution, and carbon dioxide release from the food and animal product remains would prevail. Ultimately, improper integrated and sustainable management of solid wastes would cause poor environmental hygiene and poor public health including the eruption of communicable diseases such as malaria, cholera, typhoid/paratyphoid, pneumonia, and bronchitis illnesses just to mention a few.

This prompted an investigation on how effective the environmentally solid waste management measures were implemented at the Dodoma City market places and whether they were sustainable city solid waste management-oriented or not. The study employed an analytical case study with a mixed research approach. This was not only relevant but also timely in the rapid Dodoma city socio-economic growth and its rapid urbanization to achieve cost-effective SWM and thus assure environmental hygiene and good public health of its citizens. The study was guided by the specific objectives including to: characterize solid wastes generated at the Majengo market; assess level awareness of vendors on solid waste management and identify techniques used to handle solid wastes generated at the Majengo market in Dodoma City, Tanzania.

### The scope of the study

The current study was limited at the Majengo market in Dodoma city and preferences for improving market solid waste management options only in the market premises. Furthermore, the study investigated solid wastes management oly at the Majengo market, Dodoma city Tanzania. Therefore, based on the scope of the study, its findings were only concluded among vendors who were sampled from Majengo market at Dodoma city.

## Methods

### Study design/approach

The current study employed an exploratory descriptive case study with mixed research approaches. Case study research design is recommended when a researcher intends to get detailed qualitative accounts to explore or describe the data in a real-life environment and explain the complexities of real-life situations which may not be captured through experimental or survey research [17–19]. This study design helped the researcher to intensively study SWM measures implementation among vendors at the Majengo market.

The quantitative research approach was used to guide a researcher of this study to collect information from the study respondents at a single point of time and involve a large number of respondents, to have a room of explaining results in logic, numbers, detailed, and generalized reasons in the market basis. Moreover, the study employed a qualitative research approach that enabled the researcher to gain a greater and in-depth understanding of a studied phenomenon, and experiences of the study respondents on the topic under study.

### Study population

This study involved all registered vendors and leaders of the Majengo market in Dodoma City, Tanzania who consented to join the study with an exception of the sick ones and all who were not available during the study. The market had approximately 10,000 registered vendors by the time of this study based on the report given by the market leaders. Moreover, it was reported that some vendors (the exact number was not known by the market leader by that time) were not available all the time due to the availability of commodities they sell at the market. None of the vendors was reported to be absent during the period of this study due to either sickness or other excuse. Therefore, the sampled population was drawn from approximately 8733 vendors who were reported to be present during the date of this study.

### Sampling procedure

Sampling is a process of selecting several persons or objects from a population such that the selected group contains elements representative of the characteristics found in the whole group [19]. Purposive sampling technique was used to select the region and district, simple random sampling by the lottery method to select one out of three markets in Dodoma city and convenient sampling method to select the study respondents.

### Sample size determination

A sample is referred to as a selected portion of the individuals or items that represents the aggregate of the population for the study [20]. The sample size was calculated based on the formula for single population proportion and the overall minimum sample size was determined using a single population, Proportion calculation formula:

1 $$ \mathrm{n}=\frac{{\mathrm{z}}_{\alpha }/2\ \mathrm{p}\ \left(1-\mathrm{p}\right)}{{\mathrm{d}}_2} $$

Where n = minimum sample size required for the study

- ❖ Zα/2 = 1.96, standardized normal distribution curve value for the 95% Confidence Interval
- ❖ *p* = 0.5 (assumption used in the absence of a similar previous study and to achieve the maximum possible sample size).
- ❖ d = 0.05 degree of margin of error
- ❖ n = the number of respondents to be interviewed i.e. sample size of the study
  - But the total population was less than 10,000, thus the following formula was used
  - n = no/1 + no/N

**n = 196**

Therefore, the minimum sample size of the current study was 196 respondents.

### Data collection methods/tools

Data collection tools (Questionnaires and an Interview guide), which were used in this study were developed by benchmarking from questionnaires, which were used in previous studies [13, 20, 21] and pretested by the researcher before actual field data collection. A self-administered semi-structured questionnaire on awareness status about solid waste, and awareness status about SWM, which consisted of four parts, was used to collect information from the study respondents. Structured questions had ‘Yes/No’ responses while unstructured questions provided an opportunity for the study respondent to express themselves in writings. The first part was about the demographic characteristics of the study respondents with four items including age, gender, education level, and duration of doing business at the market.

The second part consisted of 20 items about awareness on solid waste, which had ‘Yes/No’ responses. Awareness of something was described in the current study as one of the important key factors of motivating people to develop a positive or negative attitude towards an intervention (in this case solid waste management). Moreover, it was the state of the market vendors were informed about their environmental responsibilities and what was going on about the SW generation, storage, collection, transportation, treatment, disposal and the approaches, which were used to handle SW in the market.

The ‘Yes’ response weighed 1 score indicating that the respondent was aware of the item and ‘No’ weighed 0 scores indicating she/he was not. In this regard, the tool had 20 scores if all the responses would be ‘Yes’, with a cut-off point of ten scores. Respondents who scored less than 10 were defined as not aware of SWM in the market place otherwise, aware. Example of items asked in the questionnaire to assess awareness status of the respondents about solid waste included: ‘Have you ever heard about solid waste in your life? i) Yes, ii) No’; ‘Have you ever seen a solid waste in your life: i) Yes ii) No; iii) Have you ever participated in cleaning the market premises? i) Yes; iii) No.’

The output technique under the commercial waste material flow method by Tchobanoglous and Kreith [22] was used to characterize solid wastes in terms of their generation as well as discards. The approach is used to provide information regarding the condition of the waste components before separation or disposal. The researcher with the support and escort of the market leaders went to the solid waste storage and collection center located within the market premise (just along the road) to sample, sort, characterize, and quantify solid waste. The container that was used to store the waste was found full and uncovered, thus, it was easy for the researcher to see the stored waste the way they were collected (original state).

The researcher then considered and estimated the solid waste characteristics in terms of volume (to determine the rate of solid waste generation) and varieties (to identify the types of generated solid waste). The researcher worn protective gears available at the market and used a solid waste characterization checklist (developed by the researcher) based on the output technique and started to characterize a sample of drawn solid waste from the container (solid waste collectors helped to take a bundle of wastes from the container). The market leaders’ experience about the approaches they used to characterize solid waste at the market and the use of available market-based approximation tools, which were used in the market to record the amount and types of waste helped, the researcher to characterize solid waste at the market.

The third part concerned about the status of solid waste collection, transportation and disposal (SWM) (8 items). Examples of items in this part included: ‘Are there any solid waste management services provided in the market? i) Yes, ii) No’; Who offers solid waste management services? Municipality i) Yes ii) No; Community-based organizations/groups i) Yes ii) No; vendors themselves i) Yes ii) No; Private organizations/groups i) Yes ii) No; I am not aware of it i) Yes ii) No. The ‘Yes’ response weigh 1 score indicating that the issue that was questioned in the item was present and ‘No’ weighed 0 scores indicating it was not there in the market. Each study respondent answered the same questions and the process of filling them lasted between 5 to 10 min.

An in-depth un-structured interview through unstructured questions in the questionnaire targeted vendors in the field of SWM at the Majengo market who were chosen randomly. The research respondents were interviewed about the existed SWM measures and the perceived factors, which influenced its implementation to get qualitative information. The interview guide was specific to the study respondents and their roles in SWM, which consisted of open-ended questions. The first part of the interview guide consisted of questions about the status of the market cleanliness, and the second part was about the solid waste management policies/measures that existed and were implemented at the market.

The researcher informed and explained to each research respondent about the aims of the study and those who gave informed consent to participate were given questionnaires to fill and interviewed about the status of market cleanliness, solid waste generation, collection, transportation, and disposal. The researcher of this study in a selected room that assured privacy and confidentiality conducted the personally (face-to-face) interview and it lasted between 10 to 25 min per each study respondent.

### Validity

The researcher of this study used the triangulation method (semi-structured Questionnaires including individual in-depth interviews) to collect data, and this was to ensure the validity of the information provided by the study respondents. The researcher of this study developed the research tools by benchmarking research tools from previous studies [13, 20, 21] before being shared with the supervisor and subject matter for professional assistance including inputs, deletion, and correction.

### Reliability

The tool was tested for the content, language accuracy, clearness, and ability of the study respondents to understand the content to assure the trustworthiness of data, transferability, and generalizability of the study finding at the market. It was pre-tested by the researcher with professional support from supervisors, business experts, and statisticians from the University of Dodoma for reliability before their actual field use. A pilot study involving 20 consented respondents was conducted at SabaSaba Market, which was a location other than the sampled study area to test the abilities of the tool for it to give the intended results. The 20 copies of questionnaires were distributed to the sampled study respondents during the pilot study after having them are seated on chairs in the provided room at the market and given a brief instruction on how to fill them. None of the items were deleted or added; rather they were grammatically corrected accordingly and proofread again post-pilot study by the supervisor, business expert, and the statistician.

Findings from the pilot study were subjected to the scale analysis by using a statistical package for the social solution (SPSS) software program version 23. No item was extracted from the scale and the Cronbach Alpha was found to be 0.73, which was statistically accepted as an indicator that the tool was reliable to be used in this study for data collection.

### Data analysis

This study involved two types of data analysis including descriptive analysis by using Statistical Product for Service Solution (SPSS v.23) software program, which was used to analyze quantitative information. Descriptive analysis was used to analyze the demographic information of the respondents. Chi-square test and cross-tabulation were used to test the relationship between categorical data. Thematic analysis, which was used to analyze qualitative data to present them into theme/quotations. Before data analysis, manual data coding and error checking were done to check for missing data, incomplete or incorrect information.

## Results

Presentation and discussion of the findings were done per the objectives of the study.

### Level of awareness on SWM among vendors

**Fig. 1:**
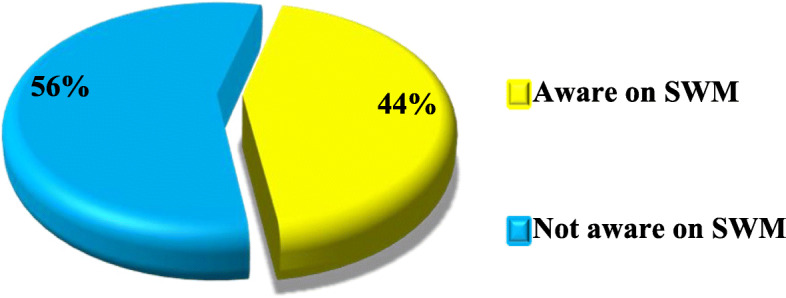
Levels of Awareness about Sustainable solid waste management measures among Vendors at Majengo market, Dodoma City Tanzania (*n* = 196) pg. 13

Awareness of something was described in the current study as one of the important key factors of motivating people to develop a positive or negative attitude towards an intervention or program. The researcher of the current study was also interested to assess the level of awareness of the vendors on solid waste management at Majengo Market in Dodoma city Tanzania. The findings showed that majority 56% (*n* = 110) of the study respondents were not aware about sustainanble solid waste management measures against a few 44% (*n* = 86) who were aware of it (Fig. 1).

During an interview, which involved individual vendors, one of them was quoted saying about their roles in SWM at the market places:

“*In our market, solid waste management has got nothing to do with our daily businesses. It is normally done by the City council and thus, they are the ones who plan and implement it, our role as the* vendor *is just paying the city SMW authority or community-based solid waste collection teams through the market leadership monthly money (Tz. shs.5,000/= to 10,000/=) for the solid waste collection services” (B34, April 2018)”*

In addition to that, one among the interviewed vendor stated that:

“*I’m less concerned about solid waste management in the market because I just come and sell goods for my domestic use and go. Those who provide SWM services to us are the ones to be responsible and accountable for it” (B156, April 2018)”*

### Factors influencing the levels of awareness on sustainable solid waste Management measures at Majengo Market, Dodoma City Tanzania

#### The relationship between age of the respondents and level of awareness on SWM

A Chi-square and cross-tabulation test was performed to find out the relationship between age groups of the respondents and their levels of awareness on the sustainable management of solid wastes at the market. Findings in Table 1 indicated that many respondents of the age group < 24 years (54.2%) were not aware of SWM as compared to other age groups. However, there was no statistically significant relationship between age groups and their levels of awareness on sustainable SWM measures at the market (*p* > 0.05) (Table 1).

**Table 1:** Relationship between Age of the respondents and the level of Awareness on SWM (*n* = 196)

Variable	Awareness on sustainable SWM measures		Chi-Square
	Aware	Not Aware	
Age			
< 24 years	49 (45.8%)	58 (54.2%)	X^2^; 0.537^a^
25–35 years	34 (41.0%)	49 (59.0%)	P-value 0.764
> 36 years	3 (50.0%)	3 (50.0%)	

#### Gender distribution of the respondents

Furthermore, the study findings in Table 2 show that majority of the study respondents who participated in this study were males 55% (*n* = 107). Despite that, findings revealed no significant relationship between being a female or male vendor could influence their levels of awareness about how to manage solid wastes at the market. Therefore, gender of vendors had little influence on SWM at the market place.

**Table 2:** Relationship between Gender of the respondents and the level of Awareness on SWM at Majengo Market, Dodoma city Tanzania (*n* = 196)

Variable	Awareness on sustainable SWM measures		Chi-Square
	Aware	Not Aware	
Gender			
Male	49 (45.8%)	58 (54.2%)	X^2^; 0.352^a^
Female	37 (41.6%)	52 (58.4%)	P-value 0.553

#### The relationship between gender of the respondents and level of awareness on SWM

Moreover, a cross-tabulation with chi-square was done to find out if there could be an existing relationship between gender of the study respondents and their levels of awareness about the sustainable management of solid wastes at the market. Findings in Table 2 point out that, gender of the respondents had no statistically significant relationship with the level of awareness about sustainable SWM measures among vendors (*p* > 0.05) (Table 2).

#### The education level of study respondents at Majengo market, Dodoma city Tanzania

Under this category, study findings indicated that, out of 196 vendors who were involved in the current study, 51% (*n* = 99) reported to have passed secondary school education followed by 79 (40%) who had primary education while 9% (*n* = 18) reported having no any formal education. Moreover, findings revealed that most vendors were educated to primary and secondary education levels as compared to those who had informal education.

#### The relationship between the education level of the respondents and the level of awareness on SWM (*n* = 196)

Analysis to find out the relationship between the education level of the respondents and their levels of awareness on SWM at the market was done by running cross-tabulation with Chi-square. Findings showed that most vendors who had a lower level of education were less aware of sustainable SWM measuresas compared to those with higher education levels. Thus, there was a statistically significant relationship between the education level of the study respondents with their levels of awareness on SWM (*p* < 0.05) (Table 3).

**Table 3:** Relationship between Education level of the study respondents and the level of Awareness on sustainable SWM measures at Majengo Market, Dodoma city Tanzania (*n* = 196)

Variable	Awareness on sutainable SWM measures		Chi-Square
	Aware	Not Aware	
Education Level			
None	3 (16.7.8%)	15 (83.3%)	X^2^; 3.152^a^
Primary education	44 (55.7%)	35 (44.3%)	*P*-value 0.035
> Secondary education	61 (61.6%)	38 (38.4%)	

#### Duration of being in the market doing business at Majengo market, Dodoma city Tanzania

In the current study, duration of doing business at the market was described to be one among the factor which could contribute to vendors' levels of awareness about sustainable solid waste management strategies and techniques. This aspect was categorized into two responses, which were the duration of below one year, in the sense that people could be less oriented and aware of the solid waste management of the market.

Another category was the duration above two years as being oriented to the market and could be aware of the solid waste management strategies and techniques. As shown in Fig. 5, study findings exposed that 73% (*n* = 144) of the respondents had a duration of above 2 years doing business at the market against the few 27% (*n* = 52) who had less than a one-year duration of doing business at the market.

#### The relationsh3ip between duration of the vendors being in the market doing the business of and their levels of awareness on SWM

A cross-tabulation with chi-square was done to determine whether there could be an existing relationship between the duration of the vendors being at the market doing business and their levels of awareness on sustainable SWM measures. Findings in Table 4 show that there was a statistically significant relationship between responds’ duration of being at the market doing businesses and their levels of awareness on SWM (*p* < 0.05) (Table 4).

**Table 4:** Relationship between duration the respondents being at Majengo Market doing the business and their levels of Awareness on sustainable SWM measures (*n* = 196)

Variable	Awareness on sustainable SWM measures		Chi-Square
	Aware	Not Aware	
Duration			
< 1 year	9 (17.3%)	43 (82.7%)	X^2^; 2.152^a^
> 2 years	84 (58.3%)	60 (41.7%)	P-value 0.041

#### The composition of solid wastes generated at Majengo market, Dodoma City Tanzania

The current study also assessed the composition of solid wastes generated at Majengo Market found in Dodoma City. This aspect was categorized into two parts, the first part sought to assess the types of solid wastes generated and the second part sought to assess the amount of solid wastes generated in the market per day.

#### Types of solid wastes generated at Majengo market, Dodoma City Tanzania

**Fig. 2:**
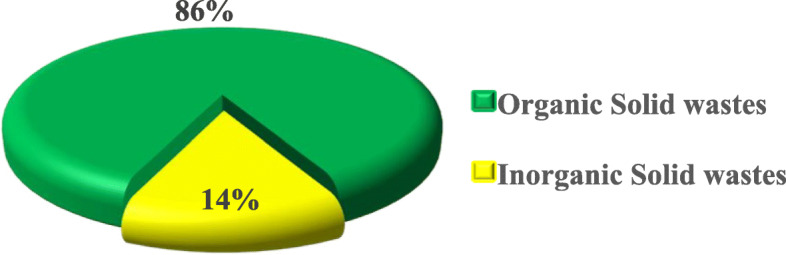
Types of solid wastes generated at Majengo Market, Dodoma City Tanzania (*n* = 196) pg. 17

The type of solid waste seen in the market was defined to represent the types of commodities being sold and bought there. These were categorized into organic solid wastes (food remains, livestock products, woods, and papers) and inorganic solid wastes (iron scrapers, plastic bags/buckets). Findings revealed that organic solid wastes were highly generated (86%) at the market than in-organic solid wastes (14%) (Fig. 2).

#### Composition of solid wastes generated at Majengo market, Dodoma City Tanzania

**Fig. 3:**
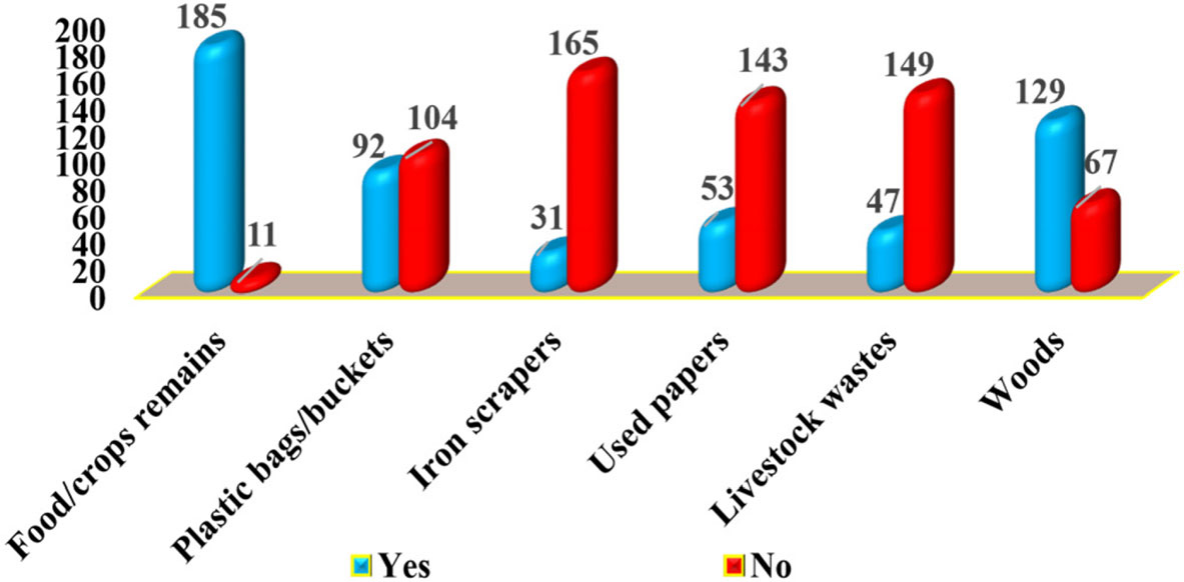
Composition of solid wastes generated at the Majengo market, Dodoma City Tanzania (*n* = 196) pg. 18

Figure 3 below indicates that the most generated organic solid wastes were food/crops remains (54.4%), followed by woods/food carrying bags 15.8%) while used papers and livestock wastes being the least (17%) and (12.8%) respectively. The most prominent in-organic solid wastes were plastic bags/buckets (49.1%) followed by iron scrapers (50.9%).

#### The rate of solid waste generation at Majengo market, Dodoma City Tanzania

The aim of the current study was also to assess the solid waste generation rate at the market. Vendors were asked questions, which intended to assess the rate to which solid wastes were generated per day. This category assessed the rate in two dimensions; the frequency of solid waste generation and how much were they generated. The frequency and volume of solid waste were measured through items in the questionnaires, which assessed the number of packaging and rotations of solid waste in the solid waste containers per day.

**Fig. 4:**
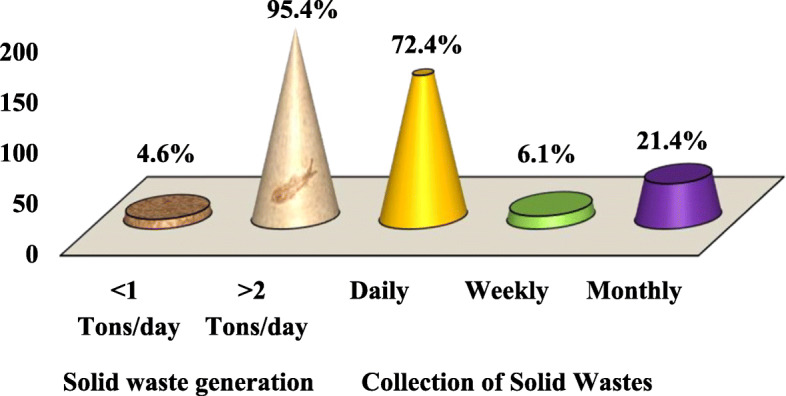
The rate and frequency of solid waste generation at Majengo Market, Dodoma City Tanzania (*n* = 196) pg. 19

Moreover, the size of the containers and trucks used to carry and transport wastes from the waste collection center within the market before being transported for treatment and disposal at the main dumpling site were used to assess frequency and volume of solid wastes at the market. 72.4% of the study respondents reported that a lot of solid wastes were generated daily as compared to 6.1% who reported solid wastes to be less generated per day while 21.4% of the study respondents reported monthly generation of solid wastes (Fig. 4). During an individual interview, one vendor reported that:

“*Solid waste generation at the market is very high to the level that, all solid waste containers can be filled in one day. The rate is contributed by the increased products which are sold and limited number of consumers who come to buy, as a result, some of the short-term consumable products/materials become spoiled and thus increase the number of wastes” (B5, April 2018)’*

Available evidence from previous studies shows that per capital, solid wastes generation estimation ranges from 0.5 to 0.8 kg/day for sub-Saharan Africa [23]. Referring to Yhdego and Kingu [12] the market waste generation in Dodoma municipal by then was 30 tons (9.8%) to 305 tons per day. The solid waste generation at Majengo Market found in Dodoma City currently was found to be > 2 tons equivalent to more than 2000kgs/day, which is remarkably very high to be generated in only one market.

#### Techniques used to handle solid wastes generated at Majengo market in Dodoma City Tanzania

Techniques and strategies used to handle solid wastes were seen to be very crucial and an indicator of the effective implementation of solid waste management policy. The current study evaluated this aspect into numerous dimensions including the collection process (frequency, means of the solid waste collection), storage (tools used to store solid wastes before disposal), and disposal strategies (services providers, expenses).

#### Solid waste collection, service provider, the frequency of SW collection and status of the Majengo market, Dodoma city Tanzania

**Fig. 5:**
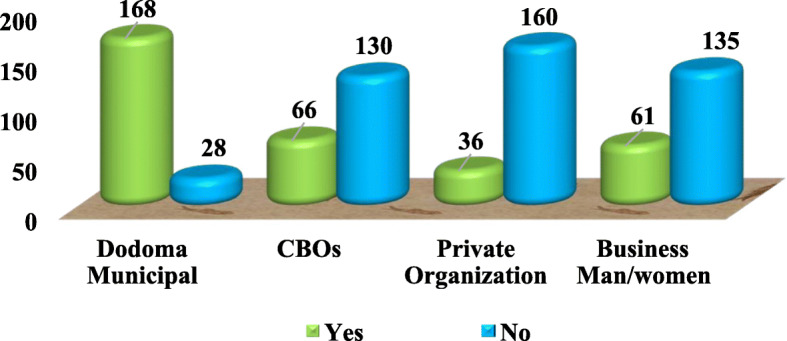
Service provider of SWM at Majengo Market, Dodoma city Tanzania (n = 196) pg. 21

The findings in Fig. 5 indicate that Municipality (85.7%) had a responsibility to provide collection services of solid wastes at the market followed by some other Community-based Organizations (CBOs) (33.7%). The CBOs were small profit-based organizations involved in the waste collection all over the city including in market areas. The existence of CBOs such as ‘Mazingira Women Group’, which reside at the Majengo ward, was acknowledged because it was also involved in solid waste collection and disposal by official contracts with the market. The organization operates based on the market schedule of waste collection and disposal especially when the city trucks were not functioning. Private organizations (18.4%) and the vendors themselves (31.1%) had a little contribution in collecting solid waste at the market.

Findings were supported by some answers, which were collected during an interview in which one vendor said:

“*Solid waste collection in the market is done by the Municipal council because we normally pay them for the service. They have a special motor vehicle that helps them to provide the service that I as an individual vendor cannot afford to do” (B99, April 2018)”*

Another vendor explained that:

“*The one who is responsible and accountable for solid waste collection in the market is the Municipal Council. Sometimes if they fail to provide the service in time, we ask Community based Organizations and sometimes ourselves to do it” (B5, April 2018)”*

Findings above are supported by the vendors’ quotes, which were noted during the interview in which one of the businesses men depicted that:

“*Collection of solid waste in the market is not good because there is an irregularity in collecting them. No specified timetable has been set and strictly followed by the City council. They just provide the service at their convenience despite the truth that we are paying for it” (B187, April 2018)”*

Additionally, another vendor was noted saying that:

“*Solid wastes are not collected in time because there is not strictly followed timetable to collect them. A lot of solid waste is accumulated in one place for long, a thing, which endangers the health of people*” (B7, April 2018)”

However, the current study found that 68.4% of the study respondents reported the frequency of solid waste collection at the market to be done at irregular timetable (no fixed schedule for solid waste collection) against 10.2% who reported it to be be done daily (10.2%) and 21.4% of the study respondents who reported solid waste to be collected weekly. Thus, the status of solid waste collection services was found to be unsatisfactory by 55.6%.

#### Tools/technique used to store solid wastes at Majengo market, Dodoma city Tanzania

**Fig. 6:**
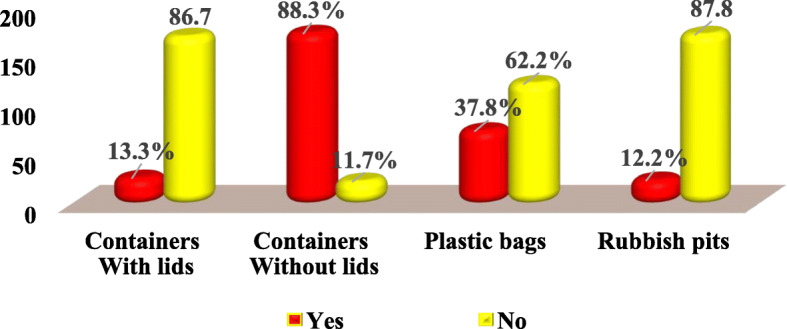
Tools/technique used to store solid wastes at Majengo Market, Dodoma city Tanzania (*n* = 196) pg. 22

The findings in Fig. 6 indicate that containers without lids (88.3%) were observed and reported to be more commonly used for storing solid wastes at the market as compared with others tools including plastic bags (37.8%), containers without lids (13.3%) and rubbish pits (12.2%). During an interview, most vendors were quoted narrating that:

“*The most used solid waste storage tool is containers without lids. They are commonly used because they are the ones, which are provided by the City council. Generally, there is a scarcity of other tools for storing solid wastes before disposal” (B120, April 2018)”*

Despite that, few vendors said that:

“*Solid wastes generated in the market are stored in containers with lids, plastic bags and sometimes if the containers are not available or they are full of wastes, we dispose them into the rubbish pits available within the market premises. This cause a large accumulation of solid wastes which at the end produce bad smell” (B77, April 2018)”*

On top of that, other vendors reported that:

“*There is a scarcity of tools/equipment which helps to store solid waste at the market. The supplied buckets or containers are not enough as compared to the rate and amount of solid wastes generated at the market. This cause a lot of solid wastes to be scattered within the market premises especially when wind or rain comes (B10, April 2018)*”

#### Solid waste disposal at Majengo market, Dodoma city Tanzania

The researcher of the current study was also interested in finding out where do the solid wastes generated at the market are disposed of. As shown in Table 5, dumpsite was the commonly used place (80.1%) for disposing of wastes as compared to other places including incinerators (1.0%) and burning (18.9%) (Table 5).

**Table 5:** Techniques used to handle solid wastes generated at Majengo Market in Dodoma City Tanzania (*n* = 196)

Variable	Frequency	Percent
**Solid Waste Collection Service Provider** **Frequency**		
Daily	20	10.2
Weekly	32	21.4
No regular timetable	134	68.4
**Total**	**196**	**100**
**Satisfaction with solid waste collection**		
Good	1	0.5
Satisfactory	86	43.9
Worse	109	55.6
**Total**	**196**	**100**
**Solid Waste Disposal Place**		
Dumpsite	157	80.1
Incinerator	2	1.0
Burning	37	18.9
**Total**	**196**	**100**

The most commonly used solid waste disposal site was observed to be at the dumpsite. Even though solid wastes were disposed at the dumpsite, some of the vendors said that:

“*Solid wastes are normally dumped at the dumpsite found within Dodoma City but the frequency of taking them for disposal is not satisfactory. The presence of improper collection and disposal of solid waste leads to the large accumulation of them at the market place. If we ask them why they do not collect wastes in time, they say the trucks that used to collect wastes sometimes become malfunctioning” (B16, April 2018)”*

#### Solid waste management strategies at Majengo market, Dodoma city Tanzania

Under this aspect, the researcher was interested to assess the presence, utilization, and effectiveness of solid waste management strategies against improper solid waste generation, collection, storage, and disposal at the market. Findings in Table 6 show that there were an existing solid waste management policy, strategies, and rules (92.9%). Despite their existence, most vendors (72.4%) reported there to be not proper tracking of people who dispose of wastes improperly in the market premises (whether market members or customers). In addition to that, most of the vendor’s opinions (82.1%) revealed that solid waste management strategies used at the market were not effective enough to control solid waste generation, collection, storage, and disposal (Table 6).

**Table 6:** Solid waste management strategies at Majengo Market, Dodoma city Tanzania (*n* = 196)

Variable	Frequency	Percentage
**Existing SWM policy, rules**		
Yes	182	92.9
No	14	7.1
**Total**	**196**	**100**
**Punishment for improper SW disposal**		
Yes	54	27.6
No	142	72.4
**Total**	**196**	**100**
**The effectiveness of the SWM policy, rules**		
Yes	35	17.9
No	161	82.1
**Total**	**196**	**100**

Most vendors were quoted during the interview saying that:

“*We heard that solid waste management strategies are existing in the market, but in fact, they are not adequately implemented. The authorized personnel are not applying them effectively and efficiently to the level people are disposing wastes improperly or wastes are spread by wind, rain or sometimes birds and other animals such as dogs to other places” (B6, April 2018)”*

Nevertheless, other vendors depicted that:

“*Solid waste management strategies are there, but they are less effective in controlling the solid waste generation, collection, storage, and disposal. Leaders do not strongly stand for them as a result, the Market is very dirty, people dispose of solid waste roughly, there are a lot of flies and bad smell around the Market” (B7, April 2018)”*

On top of that, one vendor spoke:

“*Solid waste management policy, strategies, rules, and regulations are existing in the market but they are less effective in managing the wastes. Everyone in the market can dispose of solid waste roughly. This applies to customers too; they dispose of solid wastes at any place in the market and now have turned into normal behavior” (B13, April 2018)”*

In addition to that, another vendors was quoted saying that:

“*The existing solid waste management policy, strategies, and rules used at the market are dormant because they are not strictly implemented. It could be better if they are changed or alternated with other rules, or else the leader be charged or changed regularly*” (*B15, April 2018*)”

## Discussion

### Levels of awareness on SWM among vendors at Majengo market in Dodoma City Tanzania

Findings showed that the majority of vendors who participated in this study were males, which implied that they were the ones whom most of the time engaged themselves in doing business at the market place. Based on the levels of awareness about SMW, findings of this study differ with those found by Mussa [14] and Solomon [24] who did a study on solid waste management at the households and community level. They found that people were aware of the solid waste management but only, they were not willing to pay for the service.

The findings of this study revealed that majority of vendors were not aware of SWM at the market, which impressed that, they had either little access to the information on solid waste management or fully involved in managing wastes at the market. On the other hand, their levels of awareness might either be attributed to a lack of sustainable and ongoing education programs about solid waste management at the market. The difference in the findings could be attributed to the fact that the current study was a market –based and included vendors only while others were community-based.

Needless, it was observed that being educated influenced the level of awareness on SWM among vendors and thus, the more vendors could advance their academic status, the more they could become aware of how to manage solid wastes at the market places. Therefore, education was considered as an important predictor of the level of awareness of SWM among vendors at the market. Moreover, the study findings on the duration of vendors being at the market doing their business, found that most of them were experienced in doing business at the market for more than two years and they were aware of the solid waste management measures at the market.

Thus, doing business at the market place for a long time could predict improved awareness on how to manage solid wastes among vendors in market places and the current study considered this aspect as an important predictor of the level of awareness on SWM among vendors.

### Types of solid wastes generated at Majengo market, Dodoma City Tanzania

The observed findings of the types of solid wastes entail that, most sold and bought goods at the market were consumed organic materials than inorganic ones. The findings were not surprising because the researcher studied about sustainable SWM measures specifically at the market where organic solid wastes (crops and livestock products) were sold. Nevertheless, most respondents reported that the rate of solid waste generation to be more than 2 tons per day as compared to those who reported 1 ton per day. The trend was discussed in the current study to be contributed by the increased number of vendors (approximately 2650) at the market and consumers who are served by the market as they come to sell and buy commodities.

The findings of this study do not match with those, which were found by Squire [25] who studied pollutants, and solid wastes in urban areas. It was revealed that biomedical pollutants were the major waste as compared to others. The differences could be contributed to the study settings, approaches, and population involved in the study.

### Solid waste Management at Majengo Market, Dodoma City Tanzania

The findings presented above revealed that Dodoma City Council takes a great part and largely tries to fulfill its responsibilities of making the market and other premises clean and attractive by providing waste containers and trucks to carry them to the dumpsite. The City council was responsible for solid waste collection, storage, transportation, treatment, and disposal. However, most of the vendor’s opinions revealed that solid waste management strategies used at the market were not effective enough to control solid waste generation, collection, storage, and disposal. Vendors reported scarcity of waste collection tools and vendors at the market reported the absence of fixed and regular timetable of providing those services. They also reported it to be among others, the factor leading to unsafe and inappropriate means of storing solid wastes, which would endanger the health of vendors and other people within and outside market premises.

The findings above tally with those found by Chirico [26] who assessed barriers to the sustainability of solid waste management. The findings revealed that inadequate containers to store and dispose of solid wastes, and financial constraints were the factors to improper implementation of solid waste management policy, regulations, and rules. Furthermore, despite the differences in study population and setting, Shabani [27], did a study on factors affecting community participation in solid waste management. Findings revealed that there was no proper place or tools/equipment, which was used for solid waste storage a thing that caused people to discard waste inappropriately. This is to say, failure of solid waste management also exists not only Dodoma but also elsewhere within the country.

## Conclusion

This study has been able to find that the majority of vendors were not aware of the management of solid wastes in the market. Several management factors, which were, adversely affected cleanliness: insufficient allocation of lidded containers both in number and in quality that led them to be overfilled and cause the wastes to be scattered all around the market premises. The study further observed the insufficient provision of the regular solid collection, storage, transportation and disposal services; and inadequate supervision of proper solid waste collection, storage, and transportation and disposal process. Customers were less involved in solid waste management at the market and thus they rarely have to participate to prevent improper disposal of solid waste around the market premises.

Variation in the implementation of sustainable solid waste management strategies in the sampled market could not easily be related to studied solid waste management factors. It was conditional that the implementation process was influenced by several factors apart from management factors including poor wastes segregations, inadequate market leaders and vendors’ commitments, inadequate customer involvement in the solid waste management process and the unsatisfactory availability of non-human resources.

Moreover, solid waste segregation was observed in the current study to be not performed in the market as the ever-stored SW were mixtures of different materials including metal and plastic material, food and animal remain, papers, disposable carrying bags for the purchase and other related waste materials. A source-separation of wastes was not common at the market, something that was discussed in this study to probably be the reason why varieties of SW were found mixed. Based on that, there were no tools or infrastructures available at the market, which could be used to facilitate the separation of the SW at the source.

The current study recommends that there would be regular and periodic awareness-raising mentorships sessions among vendors on the sustainable solid waste management, emphasize the solid waste management service providers to timely, and regularly provide the services to make the market clean, attractive, and safe for the well-being of vendors and consumers.

### Limitations of the study

This study was only limited to the Majengo market found at Dodoma City; therefore, the findings were not generalized to other places. Data were only collected on solid wastes thus liquid wastes data were not included during data analysis and so missing the general information about wastes management measures in Dodoma City. The financial constraint of the researcher hindering the wide coverage of data collection.

## Data Availability

Not applicable due to the involved University’s’ ethics/consent as well as participant’s confidentiality’; if required permission will first need to be sought from the University of Dodoma (UDOM).

## Abbreviations

CBOs: Community-based Organizations
CoES: College of Environmental Sciences
EMA: Environment Management Act
IRRC: Institutional Research Review Committee
NEP: National Environmental Policy
NEMC: National Environment Management Council
MSW: Management of Solid Waste
SPSS: Statistical Product for Service Solution (SPSS version.23)
TZ: Tanzania
SW: Solid Waste
SWM: Solid Waste Management
UDOM: University of Dodoma
UN: United Nations
